# Designation of a neotype for *Mazama americana* (Artiodactyla, Cervidae) reveals a cryptic new complex of brocket deer species

**DOI:** 10.3897/zookeys.958.50300

**Published:** 2020-08-11

**Authors:** Analorena Cifuentes-Rincón, Jorge Alfonso Morales-Donoso, Eluzai Dinai Pinto Sandoval, Iara Maluf Tomazella, Aline Meira Bonfim Mantellatto, Benoit de Thoisy, José Maurício Barbanti Duarte

**Affiliations:** 1 Núcleo de Pesquisa e Conservação de Cervídeos (NUPECCE), Faculdade de Ciências Agrárias e Veterinárias da Universidade Estadual Paulista (UNESP), Via de Acesso Paulo Donato Castellane, s/n CEP: 14884–900, Jaboticabal-SP, Brazil Universidade Estadual Paulista Jaboticabal Brazil; 2 Universidade Federal do Sul da Bahia, Campus Sosígenes Costa, Porto Seguro, BA, CEP: 45810–000, Brazil Universidade Federal do Sul da Bahia Porto Seguro Brazil; 3 Kwata NGO, 16 Avenue Pasteur, 97300 Cayenne, French Guiana Kwata NGO Cayenne French Guyana

**Keywords:** biodiversity, chromosomes, cytogenetics, French Guiana, mitochondrial DNA, morphology, red brocket deer, topotype

## Abstract

*Mazama americana* (red brocket deer) is the genus-type species (first species described for this genus) and the basis for the identity of other *Mazama* species. *Mazama americana* is one of the most abundant and widely distributed deer species in the neotropical forest. However, recent studies suggest that this taxon belongs to a species complex. Our goal was to collect an animal at the type locality (topotype) in French Guiana with the aim of characterizing the morphological (biometric, craniometric), cytogenetic (Giemsa, C-banding, G-banding and NOR) and molecular (mitochondrial DNA) features. The comparisons showed that the collected specimen was very similar morphologically to specimens from other South American populations, but it was cytogenetically and molecularly very different from any of the cytotypes already described for this species, corroborating the existence of a complex of cryptic species. The data suggest that the *M. americana* topotype is a different species from all the cytotypes already described in the literature and which occupy the southern region of the Amazon River. The characterization and designation of the *M. americana* neotype is the first step toward a taxonomic reorganization of the genus *Mazama*, with the potential identification of new species.

## Introduction

The genus *Mazama* Rafinesque, 1817 (Mammalia, Artiodactyla, Cervidae) has 10 species widely distributed throughout the neotropical region (Merino and Rossi 2010). However, there are controversies about the number of species and subspecies that compose the genus. Early taxonomic revisions suggested the existence of 17 species and 11 subspecies (Allen 1915), four species and 10 subspecies (Cabrera 1960), and six species and seven subspecies (Czernay 1987). However, it is suggested that morphology is not an useful tool for discrimination among species, because there is a high degree of homoplasy and morphological convergence (Grooves and Grubb 1987; Duarte et al. 2008; Merino and Rossi 2010; Gonzalez et al. 2016).

Several cytogenetically studied species have shown an extensive intraspecific polymorphism (Taylor et al. 1969; Jorge and Benirschke 1977; Neitzel 1987; Duarte and Jorge 1996; Duarte and Merino 1997; Abril and Duarte 2008; Abril et al. 2010; Valeri et al. 2018). This polymorphism would suggest a rapid speciation process driven by chromosomal rearrangements, with a diploid number of chromosomes ranging between 42 and 52, considered within six geographically established cytotypes (Duarte et al. 2008; Abril et al. 2010). As a result of this chromosomal variation, reproductive isolation has occurred in animals due to sterility problems caused by chromosomal meiosis pairing between different chromosomal lineages (Aquino et al. 2013; Cursino et al. 2014; Salviano et al. 2017). The populations in south-central South America are reproductively isolated from the Amazonian populations, being different species (Cursino et al. 2014; Salviano et al. 2017), that until now have not been described or nominated.

These results have been corroborated by mitochondrial DNA variation, which clearly demonstrates the differences between some populations of *M. americana* Erxleben, 1777 (Duarte et al. 2008, Abril et al. 2010), underlying the importance of research to characterize these taxa. Previous cytogenetic research has identified various *M. americana* cytotypes as potentially valid unamed species, and it is necessary to evaluate karyotypically previously designated names for *Mazama*. Therefore, animals should be sampled in all location types for each of the names currently positioned in the synonym of *M. americana* (Varela et al. 2010).

These taxonomic uncertainties resulted in the species being categorized by the International Union for Conservation of Nature as “deficient data”, since the identity of the current taxon had no value as an evolutionary unit (Duarte and Vogliotti 2016).

The species was described in 1777 by JCP Erxleben, a German naturalist, who is considered to be one of the founders of modern veterinary medicine. He did not collect any specimens; however, he used a series of reports by other authors as a basis for their description, mentioning the following characteristics: “*M. rufo-fufeus, ore nigro, gula alba, auriculae longitudine quatuor pollicum, oculi magni nigri, nares magnae, oris regio nigra, crura poftica longiora anticis, cauda brevis, pili breues mollesque, capitis collique fupra fufci, colli fubtus albi, corporis crurumque rufofufci, vngulae nigrae, timidiffimus, celerrimus, agilis, natat per fluuios, caro bona*”. Cayenne, in French Guiana, was considered by Erxleben as the type locality for *M. americana*.

The absence of the species holotype for a more complete morphological and genetic analysis calls for the need to describe a current topotype of the species and to propose a neotype based on it. Thus, in this work we propose a neotype for the species, as well as its morphological and genetic characterization, based on a male specimen collected in French Guiana, near the type locality of the species. In addition, we performed comparisons with the known populations of this species already studied (Duarte et al. 2008; Abril et al 2010). The comparisons demonstrate that none of them belong to the same taxon described by the type location *M. americana*, a revision of the taxonomy of this red brocket complex being required.

## Material and methods

### Obtaining the animal and samples

**Figure 1.**
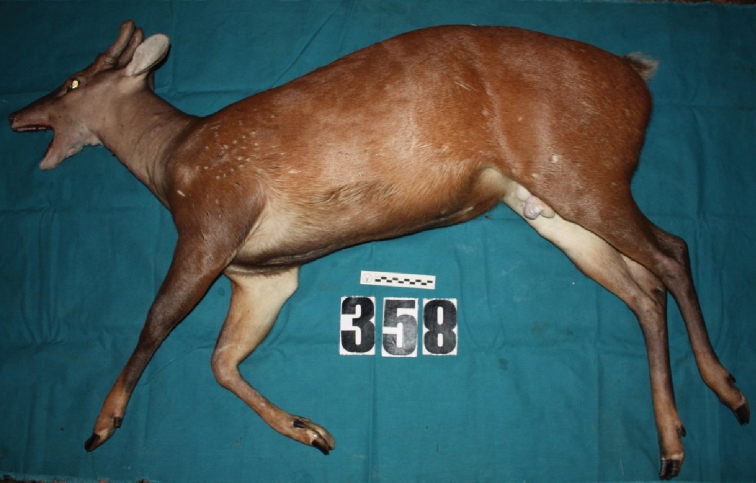
Lateral view of the adult male *Mazama americana* collected in French Guiana and proposed as neotype.

An adult male specimen of *M. americana* (Fig. 1) was collected in the city of Régina, French Guiana, 70 km from Cayenne (type locality) on 14/02/2015 by a local hunter. In French Guiana, this species is not protected by law, and can be collected without permit, the requirement being to capture outside protected areas. After collection of the individual, skin biopsies were collected and frozen in liquid nitrogen (Duarte et al. 1999) Muscle and liver samples were taken, as well as the specimen’s biometric data. The material analyzed in this study is deposited in the Museum of the Deer Research and Conservation Center (NUPECCE) – at São Paulo State University (UNESP), Jaboticabal campus, Brazil, recorded under catalog number NPC079. In accordance with the “Loi pour la reconquête de la Biodiversité” (2017) and, in compliance with Access and Benefits Sharing (above-mentioned law, titre V, article 37), a tissue sample is also kept in the collection JAGUARS, belonging to Kwata NGO, Cayenne, French Guiana, under the reference M3426_JAG.

### Biometry

Eighteen body measurements were taken using a digital caliper (0.05 mm precision) and a measuring tape. Based on these measurements, a statistical analysis of the quantitative data was performed, along with those of 41 *M. americana* individuals (adult males and females) and four individuals of different species used as an external group from the NUPECCE database through cluster analysis using the Paleontological Statistics, PAST 3.20 program (Hammer et al. 2001).

Analysis of the external morphology of the specimen was performed based on criteria used by Rossi (2000), using the photos taken immediately after collection and the entire taxidermized skin. In addition, the chromogenetic fields of the head and body were analyzed according to the nomenclature used by Hershkovitz (1982).

### Cranial morphology

Thirty-six cranial measurements were recorded using a digital caliper (0.01 mm accuracy), based on the criteria proposed by Von Den Driesch (1976). A cluster analysis using the PAST program (Hammer et al. 2001) was performed based on quantitative cranial measurements of the specimen collected and of 15 animals (male and female adults) belonging to various *M. americana* cytotypes: one *M. bororo* Duarte, 1996, two *M. gouazoubira* Fisher, 1814 and one *M. nemorivaga* Cuvier, 1817, which are stored in the NUPECCE museum (UNESP/Jaboticabal).

### Cytogenetic analysis

Metaphase chromosome slides were prepared from tissue culture (Duarte and Jorge 2003), generated through biopsy according to Verma and Babu (1995). Chromosomal preparations were subjected to conventional Giemsa staining, G-banding using trypsin digestion (Seabright 1971), C-banding by barium hydroxide solution (Sumner 1972) and Ag-RON silver nitrate staining (Howell and Black 1980). The chromosomes were classified as metacentric, submetacentric or acrocentric according to their arm relationships (Levan et al. 1964) and organized into groups according to their relative lengths (CR): Group A (large two-armed chromosomes with CR > 6%); Group C (small two-armed chromosomes with CR < 6%); Group D (large acrocentric chromosomes with CR > 5%); Group E (small acrocentric chromosomes with CR < 5%); and Group B (microcromosomes or extranumerary chromosomes with CR < 1.5%). B chromosomes were not considered in the diploid and fundamental number calculation due to the variability between metaphases of the same individual (Abril et al. 2010). Karyotypes were carried out based on the G-bands, which were used to make the schematic representation of the G-band patterns of the neotype. The cytogenetic data of the neotype were compared with the cytogenetic patterns of the *M. americana* cytotypes. We proposed the chromosomal evolution of the neotype from the hypothetical ancestor suggested by Abril et al. (2010).

### DNA extraction, amplification and sequencing

DNA was extracted from a muscle sample following the protocol of Sambrook et al. (1989). The sample was subjected to the PCR technique (Mullis et al. 1986), where two mitochondrial DNA fragments were amplified: cytochrome b (*Cyt-b*,1140 pb; Kocher et al. 1989; Duarte et al. 2008) and a control region (*D-Loop*, 690 bp; Vilà et al. 1999). DNA amplification used a final volume of 20 μl, containing 12 μl of H_2_O, 0.5 mM of dNTP, 1X of reaction buffer, 1.5 mM of MgCl_2_, 0.25 mM of each primer, 0.1 U of Taq polymerase and 3μl of DNA (15 ng/μl). The PCR protocol was 5 min at 94 °C, 35 cycles at 94 °C for 1 min, 54 °C and a final extension of 10 min at 72 °C. PCR products were submitted to 2% agarose gel electrophoresis for amplicon identification. The purification of the amplified samples followed the Dorado–Pérez (2012) protocol. Purified samples were sequenced on an automated Applied Biosystems 3730XL sequencer.

### Molecular data analysis

The sense and antisense strands of all amplified fragments of two mitochondrial genes were sequenced. The two complementary strands were aligned, thus obtaining the consensus sequence from the Clustal W program (Higgins et al. 1992) included in Bioedit (Hall 1999). The sequences obtained were organized into a matrix along with all sequences of the *M. americana* species currently published in the GenBank System world databases (Suppl. material 1: Table S1). The best molecular evolution model was selected for the data set of each gene fragment using the jModelTest v. 0.1.1 (Posada and Crandall 1998), following the corrected Akaike information criterion, AICc (Akaike 1973). Sequences for the mitochondrial *Cyt-b* gene were obtained by concatenating data from the two fragments using internal primers L14724 and H15149 for the 3’ end and FAR-L and FAR-H for the 5’end, obtaining a 1140-bp fragment.

The phylogenetic relationships between the different *M. americana* populations and the neotype were studied by Bayesian inference analysis (Huelsenbeck and Ronquist 2001), using the program MrBayes on XSEDE 3.2.6 (Ronquist and Huelsenbeck 2003), through the online program CIPRES Science Gateway (Miller et al. 2010).

Bayesian inference analyses were performed using 50,000,000 generations over four chains with two replications, adopting a 25% burn-in discard. To estimate the posterior probability, the Markov Chain Monte Carlo method was used. All trees were edited in the FigTree v. 1.4.0 program (Rambaut 2009). *Rangifer tarandus* Linnaeus, 1758, *Ozotoceros bezoarticus* Linnaeus, 1758 and *Mazama gouazoubira* were used as an external group.

## Results

### Morphology

**Figure 2.**
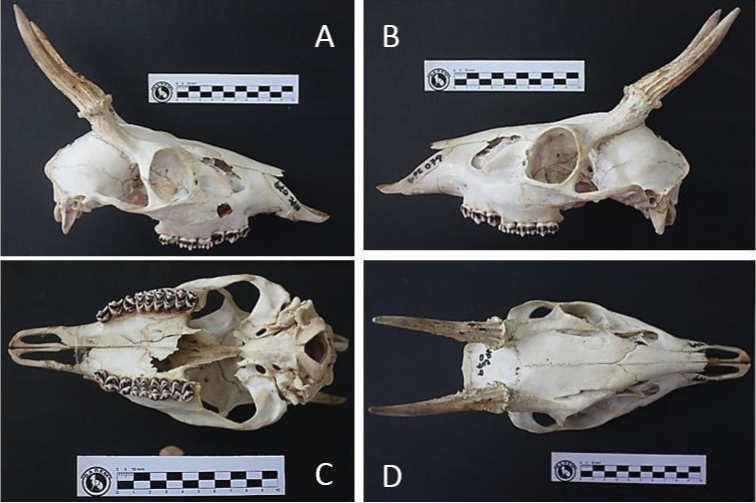
Right lateral (**A**), left lateral (**B**), ventral (**C**) and dorsal (**D**) views of the skull of the *M. americana* neotype. Scale illustrates measurement in centimeters.

The collected animal is presented in Figure 1 and the skull in Figure 2. The biometric and cranial measures are shown in the Suppl. material 2: Tables S2, Suppl. material 3: Tables S3.

Amended description of *Mazama americana* Erxleben, 1777 (Mammalia, Cervidae): Deer with predominant red coat, resembling reddish-brown brick color, gray in the face and neck region. The most intense color tone in the dorsal region. The side region the same tone as the back, slightly paler. The abdominal region the same color as the flanks, slightly brownish. Red tail in the dorsal region the same color as the back, and white in the ventral region. Whitish inguinal, buccal, gular and inner region of the ears. Black-colored posterior limbs in the caudal region, brown in the cranial region, external proximal region the same color as the lateral body region, white internal proximal region, brown external distal region, slightly reddish in the most distal portion, internal distal region also slightly brown, and the most distal portion slightly reddish. The lower and upper orbital bands slightly lighter than the rest of the face. Relatively deep lacrimal fossa. The outer ear surface lightly covered with brown hair. Smooth, varying hair lengths according to the body region, shorter and thinner hair covering the muzzle, the outer and inner surface of the ear (the latter partially hairless), the chin and the distal region of the anterior and posterior limbs. Somewhat longer hairs on the hips and tail. Strip of anteverted hair on dorsal midline of neck with more pigmented, blackened terminal band. Presence of a tuft of hair on the back of the head immediately preceding the horns. Relatively large and thick horns, dorso-caudal inclination, slightly curved and parallel to each other. Horns covered with soft tissue.

**Recording location**: Régina, French Guiana

**Collection point coordinates**: 4°19'52"N, 52°07'22"W

**Specimen deposited in**: Museum of Deer Research and Conservation Center (NUPECCE)–FCAV – São Paulo State University (UNESP) – Jaboticabal Campus.

**Classification number**: NPC079 (full skull, post skull, and taxidermized skin)

**Tissue sample deposited in**: JAGUARS collection, Kwata NGO, Cayenne, French Guiana, reference M3426_JAG

**DNA sequence deposit numbers**: MN726911 (*Cyt-b*), MN726914 (*D-Loop*)

**Karyotype**: 2n = 45 + 3Bs, FN = 51, sexual system XY_1_Y_2_

**Synonymy**: Given the cytogenetic and molecular results obtained to date, there is a high probability that several names currently synonymized with *M. americana* are valid names. Therefore, only names given to animals from French Guiana will be considered synonyms of *M. americana*, which are: *Cervus rufus* Cuvier, 1817 and *Mazama pita* Rafinesque, 1817.

**Figure 3.**
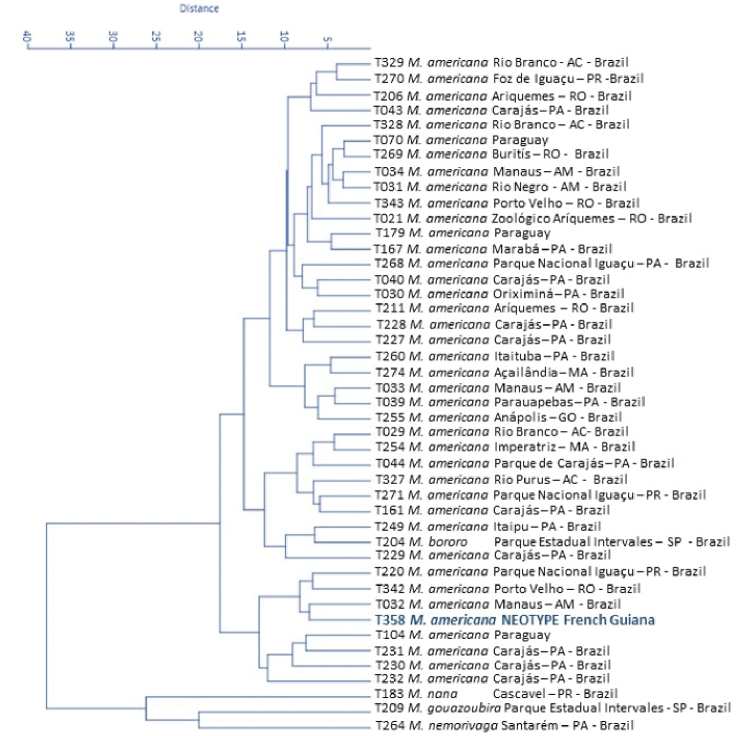
Distance tree (cluster analysis) made with biometric measurements of 39 *Mazama americana* specimens of various origins compared to the neotype and other *Mazama* species (*M. nemorivaga*, *M. gouazoubira*, *M. nana* and *M. bororo*).

**Figure 4.**
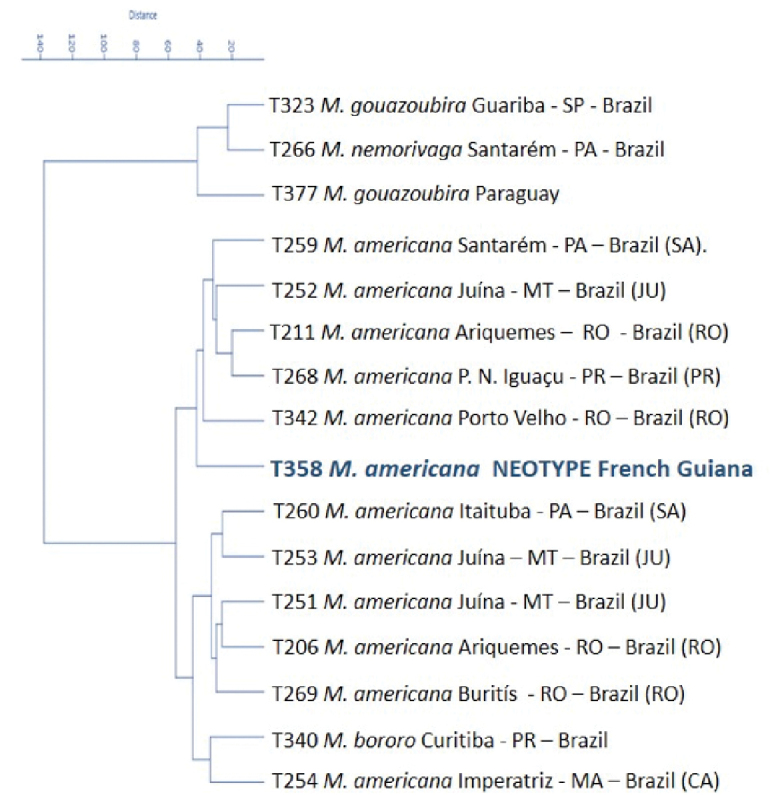
Distance tree (cluster analysis) made with cranial measurements of different *M. americana* cytotypes, in parenthesis, found in Brazil (SA-Santarém, JU-Juína, RO-Rondônia, PR-Paraná, CA-Carajás) compared to *M. americana* neotype and other *Mazama* species (*M. gouazoubira*, *M. nemorivaga* and *M. bororo*).

The results of the cluster analysis made with body measurements did not reveal morphometric differences between the distinct geographic groups of *M. americana*. The distance tree (Fig. 3) shows substantial overlap between different geographic groups. Specimens from different localities in Brazil are widely scattered across tree branches, despite known to be cytogenetically different. Some specimens of *M. americana* are superimposed on the *M. bororo* sample, showing the morphological proximity between *M. americana* and this already well-established taxon (Duarte and Jorge 2003; Vogliotti and Duarte 2009). The morphological distance tree based on cranial measurements of the individuals shows the same result, with non-differentiation between the red brocket deer specimens and cytotypes (Fig. 4).

### Cytogenetics

**Figure 5.**
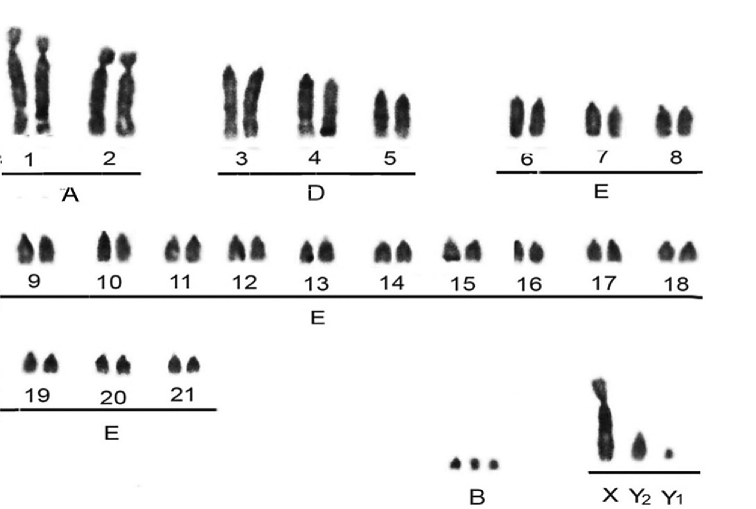
Basic karyotype belonging to the *Mazama americana* neotype (2n = 45 FN = 51 + 3Bs) under conventional Giemsa staining.

The collected animal presented a cytogenetic constitution with diploid number (2n) of 45 chromosomes and 51 chromosomal arms (fundamental number, FN). The biometric analysis classified the pairs 1 and 2 belonging to Group A; 3, 4 and 5 to Group D; and 6 to 21 to Group E. The three chromosomes classified as supernumerary or B, were acrocentric and showed no numerical variation between the metaphases analyzed. The sexual system was XY_1_Y_2_, due to an X-autosomal fusion (Fig. 5).

**Figure 6.**
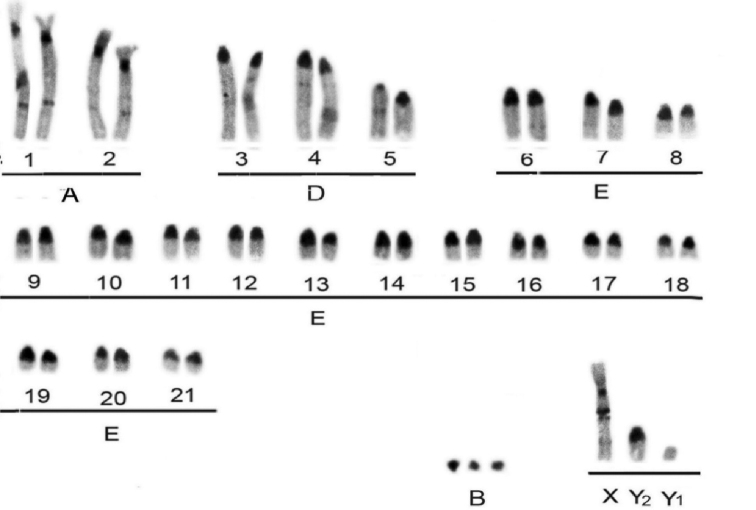
Basic karyotype belonging to the *M. americana* neotype (2n = 45, FN = 51 + 3Bs) under C-band.

Under AgNOR staining, the telomeric regions of one of the chromosomes of pair 3 and two chromosomes of pair 6 were marked. The C-band (Fig. 6) showed constitutive heterochromatin blocks in the pericentromeric region of all autosomal chromosomes, a strong interstitial heterochromatic band on the long arms of chromosomes 1 and 2, and weak bands on chromosomes 3, 4, 5, 6 and Y_2_. The X chromosome showed a large heterochromatic block in the interstitial region, near the centromere of the long arm, as well as a small and weak heterochromatic band in the terminal region of this arm. The chromosome Y_1_ is fully euchromatic and chromosomes B are heterochromatic.

**Figure 7.**
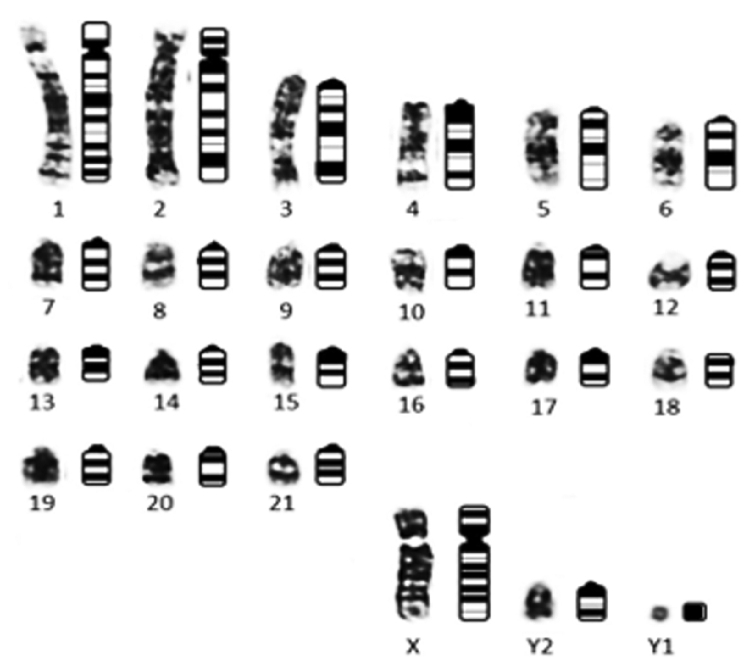
Basic karyotype belonging to the *M. americana* neotype (2n = 45 FN = 51 +3 Bs) under G-band and its schematic representation to facilitate future comparisons.

The schematic representation of the G-banding of the *M. americana* neotype is shown in Figure 7 and may be used in the future as a standard for describing new species based on the karyotype. The joint analysis of bands C and G allowed identification of the region of the homologous X chromosome to an acrocentric chromosome in Group E, thus confirming the autosomal X fusion responsible for the formation of the multiple sexual system.

### Molecular genetics

The tree generated by the analysis of the mitochondrial *Cyt-b* gene (Suppl. material 4: Figure S1) shows several *M. americana* clades, two of them with well-supported posterior probability values. These clusters have no clear geographical correlation. The first clade (posterior probability = 1) grouped animals from Paraná (southern Brazil), from Pará (northern Brazil), and Rondônia (northwestern Brazil). Another well-supported clade (posterior probability = 0.99) included the neotype with animals from Acre, Amazonas, Paraguay and Pará.

It is important to highlight that the species *M. bororo* and *M. nana* Hensel, 1872, taxonomically well-recognized and occurring in the south and southeast of Brazil, were grouped with *M. americana* from Rondônia and Juína. The distance between *M. nana* and *M. bororo* is smaller than the distance between several *M. americana* strains.

The analysis of the control region of the mitochondrial DNA *D-Loop* (Suppl. material 5: Figure S2) showed two clades: one composed of the samples of Paraná and Carajás cytotypes, together with the neotype, and another composed of the individuals from Rondônia, Juína and Jari and the *M. bororo* species. Both clades are clearly separated by a posterior probability support (posterior probability = 1).

**Figure 8.**
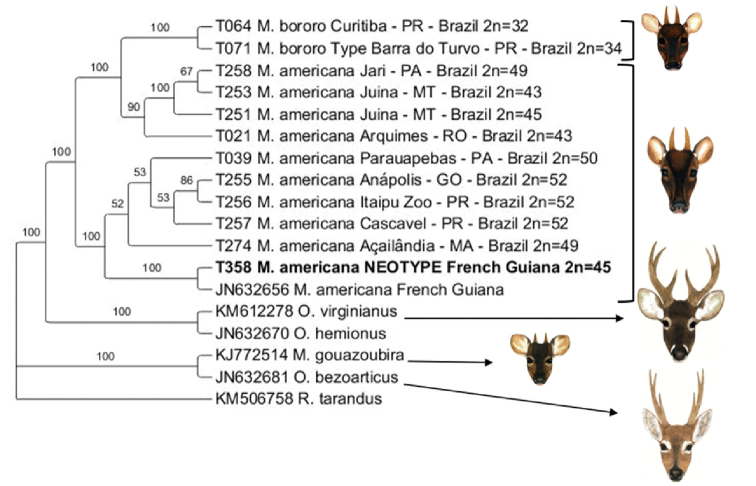
Phylogenetic tree from Bayesian inference of the fragments of the concatenated *D-Loop* and *Cyt-b* regions. The values represent the posterior probability of the analysis.

The concatenated tree of the genes *Cyt-b* and *D-Loop* (Fig. 8) follows the results obtained in the *D-Loop* analysis, with two groups, separated by high posterior probability values (1.0): one composed of animals from Paraná, Carajás and the neotype, and another clade composed of individuals from the *M. bororo* species and Rondônia, Juína and Santarém cytotypes of *M. americana*.

## Discussion

### Neotype designation

*Mazama americana* Erxleben, 1777 is the name given to a species commonly recognized for its wide distribution in the Neotropics. However, Erxleben’s description was based solely on morphology, currently considered insufficient to discriminate species of the genus *Mazama* (Duarte et al. 2008; Abril et al. 2010), due to the morphological convergence within the genus as well as the recent divergence through chromosomal changes (Gonzalez and Duarte, in press). The holotype of the species is unknown, as is any specimen of the type series, precluding any current comparison. The taxonomic complexity of the species, indicative of the existence of a complex of several cryptic species, is a *prima facie* case and can only be solved by designating a neotype (International Code of Zoological Nomenclature 1999, art. 75.3.1).

Erxleben’s (1777) original description of *M. americana* is very brief and does not include the many characteristics currently used to diagnose species of the genus *Mazama*, as he did not see any specimens but based his description on a series of reports by other authors, such as Des Marchais, 1725, who described the “*Biche de Guinee*”, as well as on a drawing of a juvenile made by Seba in 1730 called “*Cervula surinamensis*”, which simply illustrates a red deer. Linnaeus is known to have acquired specimens of Seba, but he never mentioned *Cervula surinamensis*, whose whereabouts were unknown, so it is suggested that there was no specimen that served as the basis for Erxleben’s description.

Thus, the absence of any specimen of the type series, the lack of critical information in the original description, as well as the large taxonomic uncertainties in this species complex (Abril et al. 2010; Cursino et al. 2014; Salviano et al. 2017), indicate that their identity can only be clarified by designating a neotype.

### Morphology

The genus *Mazama* is characterized by rapid diversification and morphological parallelism (Duarte et al. 2008; Gutiérrez et al. 2017), thus generating species complexes, such as in the case of *M. americana*, which present major challenges for today’s science (Abril et al. 2010; Cursino et al. 2014).

The individuals belonging to the different cytotypes of *M. americana* found in Brazil (Abril et al. 2010) and the neotype analyzed herein could not be separated in the analyses made by morphological characteristics, as already observed by Rossi (2000) and Duarte et al. (2008), suggesting great morphological similarity between the different species of the *M. americana* complex.

In the tree of morphological distances, generated from the cranial measurements of the different *M. americana* cytotypes, we found two clades, where individuals belonging to the same cytotype are positioned in both clades, thus showing there is variation in individual cranial morphometric characteristics, which do not appear to have any geographical relation. Similarly, Rossi (2000) reported the difficulty in detecting any pattern of similarity between *M. americana* samples according to cranial morphological characteristics.

The results obtained with our morphological and morphometric analyses reveal that there is morphological parallelism between *M. americana* variants, suggesting the existence of closely related characteristics, even in phylogenetically distant groups, due probably to recent diversification from their last common ancestor (Duarte et al. 2008).

### Cytogenetics

The chromosomal polymorphism found in red brocket deer is surprising and shows high levels of intra- and interspecific chromosome variation (Duarte et al. 2008; Abril et al. 2010). Thus, the neotype according to its karyotypic pattern does not fit into any of the red brocket deer cytotypes studied in Brazil (Abril et al. 2010), thus recognizing that these variants should be considered as different species according to cytogenetic evidence, since differences of more than two chromosomal pairs generate an efficient postzygotic reproductive barrier (Cursino et al. 2014; Salviano et al. 2017).

In the neotype chromosomes, the constitutive heterochromatin blocks are evident in chromosomes of Group A, weak in Group D and almost imperceptible in the first pair of Group E. According to Neitzel (1987), in chromosomes formed from tandem fusions the interstitial C bands shrink in size over time until they are so weak that they may disappear completely.

**Figure 9.**
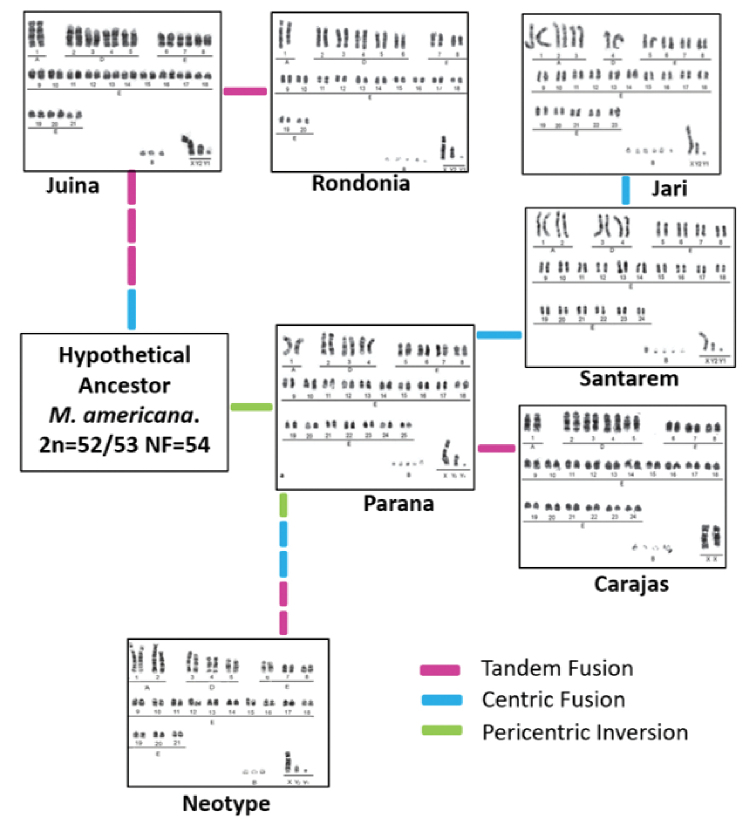
Chromosomal evolution showing the relationships of the six cytotypes compared to the *M. americana* neotype. Juine: 2n = 44/45; Rondônia: 2n = 42/43; Santarém: 2n = 50/51; Jari: 2n = 48/49; Paraná: 2n = 52/53; Carajás: 2n = 50/51; Neotype: 2n = 45 (Adapted from Abril et al. 2010).

As previously reported by Sarria-Perea (2004), cytogenetic analysis of *M. americana* cytotypes reveal an intense process of chromosomal evolution. The chromosomal reorganization is mainly due to fusions between Group D and E chromosomes to form new chromosomes and a consequent decrease in diploid number (Abril et al. 2010). According to their diploid number, it is possible to observe the approximation of the neotype to the group of individuals from Rondônia (2n = 42/43; NF = 46) and Juína (2n = 44/45; NF = 48), which have lower numbers of chromosomes. However, the chromosomal changes that occurred in the formation of Rondônia and Juína cytotypes are not the same as those involved in the karyotype formation of the *M. americana* neotype (Fig. 9), clearly showing the isolation of populations, which is also geographically confirmed, with the Amazon River in their midst acting as the major geographical barrier.

Through the use of chromosomal banding techniques, important results were generated for the karyotype study of *M. americana*. Based on their comparison, it was observed that there is a chromosomal difference between the six *M. americana* cytotypes and the neotype due to pericentric inversions, tandem fusions and centric fusions, as previously reported by Sarria-Perea (2004) and Abril et al. (2010). Based on the hypothetical ancestor of *M. americana* and the chromosomal evolution among the cytotypes proposed by Abril et al. (2010), the rearrangements involved in the karyotype formation of the neotype were two pericentric inversions, two tandem fusions and two centric fusions, thus showing greater evolutionary proximity of the neotype to the *M. americana* strain, with a higher number of chromosomes (Paraná, Santarém, Jari and Carajás; Fig. 9).

The large chromosomal variation found in *M. americana* can be explained by the theory of chromosomal fragility proposed for *Mazama* (Duarte and Jorge 1996; Vargas-Munar et al. 2010; Tomazella et al. 2017), which would induce the occurrence of breakages and chromosomal exchanges. These chromosomal rearrangements could have led to the formation of new species in a relatively short time after geographical isolation, given that chromosomal changes can promote incipient divergence and lead ultimately to species diversification (Potter et al. 2017).

Consequently, it is clear that the proposed neotype does not belong to the same species as the known *M. americana* cytotypes (Duarte et al. 2008; Abril et al. 2010), since the number of chromosomal pairs involved in these differences certainly generate an insurmountable postzygotic reproductive barrier due to sterility of the hybrid, as evidenced by Salviano et al. (2011) and Cursino et al. (2014) in the same specific complex. Thus, cytogenetics is the more important characteristic for the reclassification of the individuals of the group at the species level. With *M. americana* of Cayenne as the first description (Erxleben 1777), it is now necessary to redescribe and name all other species that are different from this neotype.

### Molecular phylogeny

The result of the concatenated analysis of the two *mtDNA* fragments (*Cyt-b* and *D-Loop*), broadly followed the results obtained from the analysis of the separate genes, showing at least two evolutionary units for *M. americana*, yielding results very similar to previous studies (Carnelossi 2008; Duarte et al. 2008; Abril et al. 2010), but complemented now by information of the neotype. However, these analyses make clear the numerous gaps in taxonomic and evolutionary knowledge of the *M. americana* complex.

The specimen of *M. americana* analyzed in this study complies with all the conditions required by the International Code of Zoological Nomenclature (1999) in force to be considered as neotype. The proposal of a *M. americana* neotype based on the detailed description of a current topotype opens great possibilities for describing new species within the genus *Mazama*. At this time, since there is a *M. americana* pattern (neotype), it is possible to define where the current cytotypes will be positioned. It is necessary that the entire nomenclature assigned to *Mazama* be reviewed from a cytogenetic point of view. For this, it would be necessary to access the chromosomal pattern for each of the available names. This can only be achieved if current topotypes are collected to define their karyotypes and position them taxonomically. In addition, molecular analysis from type series in museums could be used to help clarify the taxonomy (Gutiérrez et al. 2017)

It should be reiterated that it remains a formidable challenge to resolve the relationships between recently separated species, as in, for instance, the case of *M. americana*. However, this study has been able to confirm the existence of different species within the *M. americana* complex, as previously proposed by Duarte et al. (2008), Carnelossi (2008), Abril et al. (2010), Cursino et al. (2014) and Salviano et al. (2017), since until now the neotype does not match with any known cytotype.

This is the first reference available in the literature regarding the establishment of a neotype for *M. americana*, which is the starting point for the description of new species and possible change in the nomenclature of the genus *Mazama*.

## Supplementary materials

Supplementary material 1 Table S1. Samples used in this study for genetic analysis Data type molecular data Explanation note Nupecce’s identification, species, gene accessed, accession number in genbank, origin (locality) and source (when collected). https://binary.pensoft.net/file/441294This dataset is made available under the Open Database License (http://opendatacommons.org/licenses/odbl/1.0/). The Open Database License (ODbL) is a license agreement intended to allow users to freely share, modify, and use this Dataset while maintaining this same freedom for others, provided that the original source and author(s) are credited.Analorena Cifuentes-Rincón, Jorge Alfonso Morales-Donoso, Eluzai Dinai Pinto Sandoval, Iara Maluf Tomazella, Aline Meira Bonfim Mantellatto, Benoit de Thoisy, José Maurício Barbanti Duarte

Supplementary material 2 Table S2. Biometric data of the *M. americana* neotype Data type species data Explanation note Measured in cm and mass in kg. https://binary.pensoft.net/file/441295This dataset is made available under the Open Database License (http://opendatacommons.org/licenses/odbl/1.0/). The Open Database License (ODbL) is a license agreement intended to allow users to freely share, modify, and use this Dataset while maintaining this same freedom for others, provided that the original source and author(s) are credited.Analorena Cifuentes-Rincón, Jorge Alfonso Morales-Donoso, Eluzai Dinai Pinto Sandoval, Iara Maluf Tomazella, Aline Meira Bonfim Mantellatto, Benoit de Thoisy, José Maurício Barbanti Duarte

Supplementary material 3 Table S3. Cranial measurements of *M. americana* neotype represented in millimeters Data type species data https://binary.pensoft.net/file/441296This dataset is made available under the Open Database License (http://opendatacommons.org/licenses/odbl/1.0/). The Open Database License (ODbL) is a license agreement intended to allow users to freely share, modify, and use this Dataset while maintaining this same freedom for others, provided that the original source and author(s) are credited.Analorena Cifuentes-Rincón, Jorge Alfonso Morales-Donoso, Eluzai Dinai Pinto Sandoval, Iara Maluf Tomazella, Aline Meira Bonfim Mantellatto, Benoit de Thoisy, José Maurício Barbanti Duarte

Supplementary material 4 Figure S1. Phylogenetic tree of the Cyt-b gene Data type phylogenetic tree Explanation note Bayesian Inference (BI) Analysis. The values represent the posterior probability of the analysis. External group: *R. tarandus*, *M. gouazoubira* and *O. bezoarticus*. https://binary.pensoft.net/file/441297This dataset is made available under the Open Database License (http://opendatacommons.org/licenses/odbl/1.0/). The Open Database License (ODbL) is a license agreement intended to allow users to freely share, modify, and use this Dataset while maintaining this same freedom for others, provided that the original source and author(s) are credited.Analorena Cifuentes-Rincón, Jorge Alfonso Morales-Donoso, Eluzai Dinai Pinto Sandoval, Iara Maluf Tomazella, Aline Meira Bonfim Mantellatto, Benoit de Thoisy, José Maurício Barbanti Duarte

Supplementary material 5 Figure S2. Phylogenetic tree of the *D-Loop* gene Data type phylogenetic tree Explanation note Bayesian inference (BI) Analysis. The values represent the posterior probability of BI. External group: *O. bezoarticus*, *R. tarandus*, *M. gouazoubira*. https://binary.pensoft.net/file/441298This dataset is made available under the Open Database License (http://opendatacommons.org/licenses/odbl/1.0/). The Open Database License (ODbL) is a license agreement intended to allow users to freely share, modify, and use this Dataset while maintaining this same freedom for others, provided that the original source and author(s) are credited.Analorena Cifuentes-Rincón, Jorge Alfonso Morales-Donoso, Eluzai Dinai Pinto Sandoval, Iara Maluf Tomazella, Aline Meira Bonfim Mantellatto, Benoit de Thoisy, José Maurício Barbanti Duarte
