# Supplementary material for: Designation of a neotype for Mazama americana (Artiodactyla, Cervidae) reveals a cryptic new complex of brocket deer species
Source: Zookeys. 2020 Aug 11;958:143–64. doi: 10.3897/zookeys.958.50300 (PMC7434805; doi:10.3897/zookeys.958.50300)
Supplement: Supplementary material 1 — Table S1. Samples used in this study for genetic analysis [file zookeys-958-143-s001.pdf]

SUPPLEMENTARY MATERIAL TABLE S1

Table S1. Samples used in this study for genetic analysis. Nupecce's identification, species, gene accessed, accession number in genbank, origin (locality) and source (when collected).

ID. Nupecce	Species	Gene	Accession number	Origin/Born in	source
T16	*Mazama americana*	*Cyt-b*	DQ789209	Cuiabá-MT. Brazil	Captivity
T18	*Mazama americana*	*Cyt-b*	DQ789211.2	Vilhena-RO. Brazil	Captivity
T21	*Mazama americana*	*Cyt-b /D-loop*	DQ789216.2/GU305930	Ariquemes-RO. Brazil	Captivity
T22	*Mazama americana*	*Cyt-b*	DQ789217	Arquimes-RO. Brazil	Captivity
T28	*Mazama americana*	*Cyt-b*	DQ789218.2	Rio Branco-AC. Brazil	Captivity
T31	*Mazama americana*	*Cyt-b*	DQ789219.2	Alto Rio Negro-AM. Brazil	Captivity
T34	*Mazama americana*	*Cyt-b*	DQ789220.2	Manacapuru-AM. Brazil	Captivity
T35	*Mazama americana*	*Cyt-b*	DQ789221.2	Belém Zoo-AM. Brazil	Captivity
T36	*Mazama americana*	*Cyt-b*	DQ789222.2	Projeto Jari-PA. Brazil	Captivity
T39	*Mazama americana*	*Cyt-b*	DQ789223	Parauapebas-PR. Brazil	Captivity
T40	*Mazama americana*	*Cyt-b*	DQ789224.2	Carajás-PA. Brazil	Captivity
T41	*Mazama americana*	*Cyt-b*	DQ789225.2	Carajás Zoo-PA. Brazil	Captivity
T70	*Mazama americana*	*Cyt-b*	DQ789230.0	Ciudad del Este-Paraguay	Captivity
T110	*Mazama americana*	*Cyt-b*	DQ789201.2	Terra boa-PR. Brazil	Captivity
T120	*Mazama americana*	*Cyt-b*	DQ789204.2	Unknown	Captivity
T192	*Mazama americana*	*Cyt-b*	DQ789212	Unknown	Captivity
T161	*Mazama americana*	*Cyt-b*	DQ789207	Carajás-PA. Brazil	Captivity
T164	*Mazama americana*	*Cyt-b*	DQ789208.2	Carajas-PA. Brazil	Captivity
T205	*Mazama americana*	*Cyt-b / D-loop*	DQ789215.2/GU305922	Foz do Iguaçu-PR. Brazil	Captivity
T211	*Mazama americana*	*D-loop*	GU305931	Arquimes-RO. Brazil	Captivity
T247	*Mazama americana*	*D-loop*	GU305934	Juina-MT. Brazil	Captivity
T248	*Mazama americana*	*D-loop*	GU305935	Juina-MT. Brazil	Captivity
T251	*Mazama americana*	*D-loop*	GU305933	Juina-MT. Brazil	Captivity
T253	*Mazama americana*	*Cyt-b/D-loop*	MN726908/GU305937	Juina-MT. Brazil	Captivity
T254	*Mazama americana*	*D-loop*	GU305926	Imperatriz-MA. Brazil	Captivity
T255	*Mazama americana*	*Cyt-b/D-loop*	MN726909/GU305927	Anápolis-GO. Brazil	Captivity
T256	*Mazama americana*	*D-loop*	GU305924	Itatipu Zoo-PR. Brazil	Captivity
T257	*Mazama americana*	*D-loop*	GU305925	Cascavel-PR. Brazil	Captivity
T258	*Mazama americana*	*Cyt-b/D-loop*	MN726910/ GU305938	Santarém/Jari-PA. Brazil	Captivity
T259	*Mazama americana*	*D-loop*	GU305929	Santarém-PR. Brazil	Captivity
T260	*Mazama americana*	*D-loop*	GU305939	Itaituba-Pará. Brazil	Captivity
T269	*Mazama americana*	*D-loop*	GU305932	Buritis-RO. Brazil	Captivity
T274	*Mazama americana*	*D-loop*	GU305928	Açailândia-MA. Brazil	Captivity
T358	*Mazama americana*	*Cyt-b/D-loop*	MN726911/MN726914	Reginá. French Guiana	Wild
------	*Mazama americana*	*Cyt-b/D-loop*	JN632656	French Guiana	------
T02	*Mazama nana*	*Cyt-b*	DQ789214.2	Iguazu. Paraguay	Captivity
T51	*Mazama nana*	*Cyt-b*	DQ789227	Curitiba Zoo-PR. Brazil	Captivity
T064	*Mazama bororo*	*D-loop*	MN726912	Curitiba-PR. Brazil	Captivity
T071	*Mazama bororo*	*Cyt-b/D-loop*	DQ789231.2/MN726913	Barra do Turvo-PR, Brazil	Captivity
T072	*Mazama bororo*	*Cyt-b*	MG786263.1	Barra do Turvo-PR, Brazil	Captivity
T314	*Mazama gouazoubira*	*Cyt-b/D-loop*	KJ772514	Pantanal-MS. Brazil	Wild
------	*Odocoileus virginianus*	*Cyt-b/D-loop*	KM612278	-----------------------------	-----

------	*Odocoileus hemionus*	*Cyt-b/D-loop*	JN632670	------------------------------	-----
------	*Ozotoceros bezoarticus*	*Cyt-b/D-loop*	JN632681	------------------------------	-----
------	*Rangifer tarandus*	*Cyt-b/D-loop*	KM506758	------------------------------	-----
