# Supplementary material for: Designation of a neotype for Mazama americana (Artiodactyla, Cervidae) reveals a cryptic new complex of brocket deer species
Source: Zookeys. 2020 Aug 11;958:143–64. doi: 10.3897/zookeys.958.50300 (PMC7434805; doi:10.3897/zookeys.958.50300)
Supplement: Supplementary material 2 — Table S2. Biometric data of the M. americana neotype [file zookeys-958-143-s002.pdf]

SUPPLEMENTARY MATERIAL TABLE S2

Table S2. Biometric data of the *M. americana* neotype. Measured in cm and mass in kg.

Parameters	Measurements	Parameters	Measurements
Right antler length	2,35 cm	Neck circumference	36,00 cm
Left antler length	2,5 cm	Body length	96,00 cm
Right antler diameter	2,35 cm	Thorax circumference	78,00 cm
Left antler diameter	2,5 cm	Abdomen circumference	91,50 cm
Between antlers	2,11 cm	Tail length	12,50 cm
Ear length	10,50 cm	Height	71,00 cm
Between eyes	6,00 cm	Metacarpus length	16,00 cm
Head length	27,00 cm	Metatarsus length	26,50 cm
Head width	10,00 cm	Mass	44,6 Kg
