# Supplementary material for: Designation of a neotype for Mazama americana (Artiodactyla, Cervidae) reveals a cryptic new complex of brocket deer species
Source: Zookeys. 2020 Aug 11;958:143–64. doi: 10.3897/zookeys.958.50300 (PMC7434805; doi:10.3897/zookeys.958.50300)
Supplement: Supplementary material 3 — Table S3. Cranial measurements of M. americana neotype represented in millimeters [file zookeys-958-143-s003.pdf]

**SUPPLEMENTARY MATERIAL TABLE S3**

**Table S3.** Cranial measurements of *M. americana* neotype represented in millimeters.

| | | | | | | | |
|---|---|---|---|---|---|---|---|
| TL | *223,30* | *LMR* | *36,36* | *LR* | *171,53* | *LFB* | *67,40* |
| CBL | 211,15 | LPR | 30,33 | LP | 213,98 | GBAO | 67,00 |
| BL | 197,73 | GILO | 35,43 | AK | 151,70 | LBBO | 52,05 |
| SSL | 128,75 | GIHO | 36,43 | GLN | 70,53 | ZB | 101,86 |
| PR | 70,73 | GMB | 63,75 | SLFL | 118,17 | GBAN | 25,68 |
| BCA | 39,79 | GBOC | 41,50 | OPL | 99,66 | GBAP | 30,38 |
| BFA | 160,84 | GBBP | 57,15 | LLP | 66,38 | GPB | 41,39 |
| VL | 117,59 | GBFM | 20,02 | LCR | 65,73 | BHPSN | 51,03 |
| MFL | 115,94 | HFM | 18,49 | LN | 104,15 | GNB | 63,42 |

TL= total length, CBL= condilobasal length, BL= basal length, SSL= short skull length, PR= premolare – prostion, BCA= basecranial axis, BFA= basefacial axis, VL= viscerocranium length, MFL= median frontal length, LN= lambda – nasal, LR= lambda – Rhinion, LP= lambda – prostion , AK= akrokranium, GLN= greatest length of the nasals, SLFL= short lateral facial length, OPL= oral palatal length, LLP= lateral length of the premaxilla, LCR= length of the cheektooth row, LMR= length of the molar row, LPR= length of the premolar row, GILO= greatest inner length of the orbit, GIHO= greatest inner height of the orbit, GMB= greatest mastoid breadth, GBOC= greatest breadth of the occipital condyles, GBBP= greatest breadth at the bases of the paraoccipital, GBFM= greatest breadth of the foramen magnum, HFM= Height of the foramen magnum: Basiom - Opisthion, GNB= Greatest neurocranium breadth, LFB= Least frontal breadth, GBAO= greatest breadth across the orbits, LBBO= least breadth between the orbits, ZB= zygomatic breadth, GBAN= greatest breath across the nasals, GBAP= greatest breadth across the premaxillae, GPB= greatest palatal breadth, BHPSN= Basion – The highest point of the superior nuchal crest, CNC= capacidade neucraniana.
