# Supplementary material for: Designation of a neotype for Mazama americana (Artiodactyla, Cervidae) reveals a cryptic new complex of brocket deer species
Source: Zookeys. 2020 Aug 11;958:143–64. doi: 10.3897/zookeys.958.50300 (PMC7434805; doi:10.3897/zookeys.958.50300)
Supplement: Supplementary material 4 — Figure S1. Phylogenetic tree of the Cyt-b gene [file zookeys-958-143-s004.pdf]

SUPPLEMENTARY MATERIAL FIGURE S1

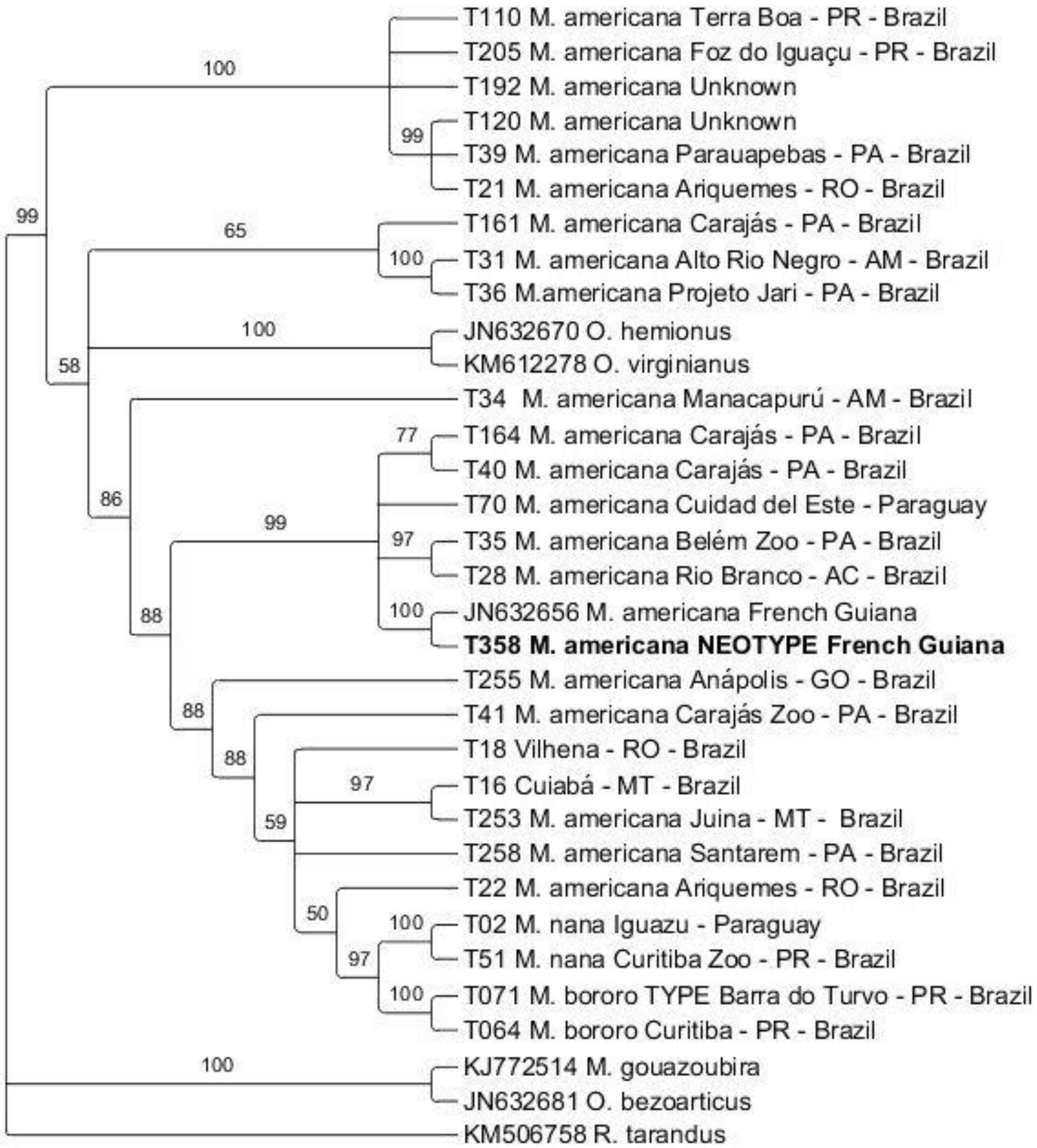

Figure S1. Phylogenetic tree of the *Cyt-b* gene. Bayesian Inference (BI) Analysis. The values represent the posterior probability of the analysis. External group: *R. tarandus*, *M. gouazoubira* and *O. bezoarticus*.
