# Supplementary material for: Designation of a neotype for Mazama americana (Artiodactyla, Cervidae) reveals a cryptic new complex of brocket deer species
Source: Zookeys. 2020 Aug 11;958:143–64. doi: 10.3897/zookeys.958.50300 (PMC7434805; doi:10.3897/zookeys.958.50300)
Supplement: Supplementary material 5 — Figure S2. Phylogenetic tree of the D-Loop gene [file zookeys-958-143-s005.pdf]

SUPPLEMENTARY MATERIAL FIGURE S2

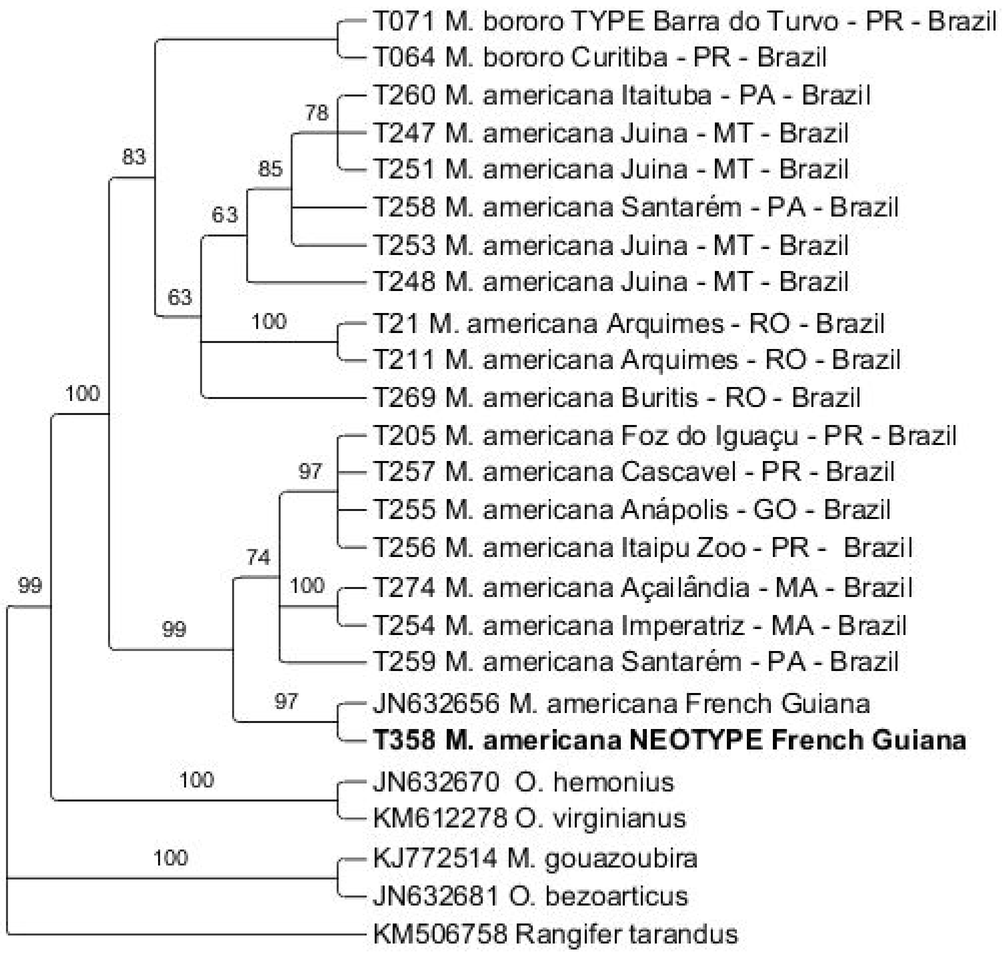

Figure S2. Phylogenetic tree of the *D-loop* gene. Bayesian inference (BI) Analysis. The values represent the posterior probability of BI. External group: *O. bezoarticus, R. tarandus, M. gouazoubira.*
